# Prognostic Value of Hyperglycemia on Admission on In-hospital Outcomes in Patients Presenting with ST-elevation Myocardial Infarction

**DOI:** 10.7759/cureus.7024

**Published:** 2020-02-17

**Authors:** Muhammad Shahid, Hafiz Muhammad Asif Zarif, Muhammad Shahzad Farid, Muhammad Shoaib Abid, Burhan Akhtar, Momin Rasheed Khan

**Affiliations:** 1 Cardiology, Ch. Pervaiz Elahi Institute of Cardiology, Multan, PAK

**Keywords:** acute st-elevation myocardial infarction, hyperglycemia, in-hospital mortality

## Abstract

Background

Type 2 diabetes mellitus (T2DM) is associated with acute coronary syndrome, and elevated blood glucose levels on hospital admission may influence outcomes in patients with ST-elevated myocardial infarction (STEMI). We conducted this study to determine the prognostic outcome of hyperglycemia at admission on in-hospital outcomes of STEMI patients with and without T2DM.

Methods

This prospective study was conducted from June 13, 2018, to October 12, 2019, and included patients older than 18 years diagnosed with STEMI. For our purposes, hyperglycemia was defined as blood glucose levels >140 mg/dl. Hypertension was considered as systolic blood pressure >140 mmHg or diastolic pressure > 90 mmHg. The predictive value of glycemia on admission for outcomes was assessed via patient mortality following thrombolysis or percutaneous coronary intervention (PCI).

Results

Our study included 256 patients (196 men, 76.5%; 60 women, 23.5%) with a mean age of 55 ± 11 years. A total of 92 patients (35.9%) were admitted with known T2DM diagnoses: 72 of them had hyperglycemia and 20 patients had euglycemia (p = 0.0001). Post-PCI mortality was six (18.8%) in the hyperglycemic group and one (2.2%) in the euglycemic group (p = 0.03). In-hospital mortality was higher in the hyperglycemic group (n = 12, 12.5%) compared to the euglycemic group (n = 6, 3.7%; p = 0.015). Significant risk factors of mortality for STEMI patients with hyperglycemia on admission were age 60 years or older (odds ratio [OR], 5.63 [1.54-20.58]; p = 0.007), heart failure on admission (OR, 6.84 [1.85-25.22)]; p = 0.003), T2DM (OR, 4.14 [0.50-33.96]; p = 0.05), and presenting with renal failure (OR, 6.78 [1.74-26.42]; p = 0.009).

Conclusion

Thrombolysis and PCI are effective and safe treatments in STEMI patients. Hyperglycemia has a great adverse impact on hospital outcomes in patients with or without T2DM. STEMI patients with hyperglycemia on hospital admission have higher mortality rates.

## Introduction

Type 2 diabetes mellitus (T2DM) is a major health problem globally. According to the World Health Organization, the prevalence of diabetes in adults has increased from 4.7% in 1980 to 8.5% in 2014 [1]. The prevalence has been increasing rapidly in developing countries like Pakistan [2]. There is a strong correlation between T2DM and acute coronary syndrome (ACS), and the prevalence rate of ACS in T2DM patients has increased from 18% in 1997 to 22.6% in 2018 [3]. The prevalence of T2DM is higher in women (26.3%) than in men (20.8%) [4]. T2DM patients with ACS have high chances of atherosclerosis, myocardial infarction, and worse outcomes compared to patients without T2DM. Patients diagnosed with ST-elevated myocardial infarction (STEMI) with hyperglycemia on hospital admission represent a greater clinical challenge in achieving outcomes normally expected following thrombolysis or percutaneous coronary intervention (PCI) [5,6].

The pathophysiological mechanisms of thrombus formation, vascular inflammation, and platelet aggregation are different in T2DM patients compared to patients without T2DM [7,8]. ACS patients with STEMI but without T2DM have higher chances of successful treatment than those with T2DM. However, the possible link between diabetes, hyperglycemia, and STEMI and early outcomes has not yet been established. We conducted this study to determine the prognostic outcome of hyperglycemia at the time of hospital admission in STEMI patients with and without T2DM.

## Materials and methods

We conducted this prospective study on STEMI patients admitted in the emergency department of Ch. Pervaiz Elahi Institute of Cardiology from June 13, 2018, to October 12, 2019. The study included patients older than age 18 years diagnosed as STEMI. The diagnostic criteria for STEMI were chest discomfort and angina lasting longer than 30 minutes, ST-elevation >0.1 mV in two adjacent electrocardiography leads, and at least one elevated biomarker (creatine kinase myocardial band, creatine phosphokinase, or troponin I).

All patients received a blood glucose test on admission. Hyperglycemia was defined as blood glucose levels >140 mg/dl. All patients underwent a routine laboratory test to identify T2DM. Hypertension was defined as systolic blood pressure >140 mmHg or diastolic pressure > 90 mmHg. Acute renal failure was defined as using progressive increase in serum creatinine ≥ 0.3 mg/dl over 48 hours.

Our study was approved by the institutional ethical research body. Patient information was kept confidential, and no identifying data were disclosed. Initially, 289 patients were evaluated, but 33 were excluded from the study population due to a lack of blood glucose values on admission. The study population consisted of 256 patients. Patients were sorted into two groups based on glucose levels on admission: a hyperglycemia group (with blood glucose >140 mg/dl) and a euglycemic group (with blood glucose >140 mg/dl). Outcomes were assessed via the mortality rate following treatment with thrombolysis or PCI.

Study data were analyzed using IBM SPSS Statistics for Windows, Version 23.0 (Armonk, NY: IBM Corp.), and quantitative variables were calculated as mean ± standard deviation. Categorical variables were calculated as frequency and percentage. To compare the groups, we used the χ2 test and unpaired sample t-tests. P-values <0.05 were considered statistically significant.

## Results

Patient demographics and baseline clinical information is presented in Table 1. Our study included 256 patients (196 men, 76.5%; 60 women, 23.5%) with a mean age of 55 ± 11 years. The hyperglycemia group contained 96 patients (75 men, 78.13%; 21 women, 21.87%) and the euglycemic group contained 160 patients (121 men, 75.63%; 39 women, 24.37%; p = 0.03). Eighty-six patients (29.7%) had hypertension. A total of 92 patients had known T2DM on admission: 72 patients were in the hyperglycemic group and 20 were in the euglycemia group (p = 0.0001). The mean duration of T2DM in the study population was 6.2 ± 3.8 years. Heart failure on admission was more prevalent in the hyperglycemic group (28.12%) as compared to the euglycemic group (18.75%; p = 0.11), indicating an association of hyperglycemia with heart failure.

**Table 1:** Demographic and baseline clinical data CAD, coronary artery disease; SD, standard deviation.

Characteristic	Population (N = 256)	Hyperglycemia group (n = 96)	Euglycemia group (n = 160)	P-value
Age ± SD (years)	55 ± 11	55 ± 10	54 ± 12	0.30
Gender ratio, male (%)/female (%)	196 (76.5%)/ 60 (23.5%)	75 (78.13%)/ 21 (21.87%)	121 (75.63%)/ 39 (24.37%)	0.03/0.76
Hypertension (%)	86 (29.7%)	36 (37.5%)	50 (31.25%)	0.37
Diabetes (%)	92 (35.93%)	72 (75%)	20 (12.5%)	0.0001
Smoker (%)	168 (65.6%)	54 (56.25%)	104 (65.0%)	0.20
History of CAD (%)	30 (11.7%)	12 (12.5%)	18 (11.25%)	0.92
Heart failure on admission (%)	57 (22.26%)	27 (28.12%)	30 (18.75%)	0.11
Cardiogenic shock (%)	5(1.9%)	2 (2.08%)	3 (1.8%)	0.72
Acute renal failure (%)	24 (9.3%)	13(13.54%)	11 (6.8%)	0.11

Patients were treated with thrombolysis or primary PCI. Thrombolytic treatment was given to 27 patients in the hyperglycemia group (28.1%) and 75 patients in the euglycemic group (46.8%; p = 0.004). Primary PCI was performed in 33 patients in the hyperglycemia group (34.3%) and 45 patients in the euglycemia group (28.12%; p = 0.36). Post-PCI mortality was six (18.8%) in the hyperglycemic group and one (2.2%) in the euglycemia group (p = 0.03). Overall in-hospital mortality was higher in the hyperglycemic group (n = 12, 12.5%) than in the euglycemic group (n = 6, 3.7%; p = 0.015; Table 2).

**Table 2:** Therapeutic treatment and outcomes PCI, percutaneous coronary intervention.

Characteristic	Population (N = 256)	Hyperglycemia group (n = 96)	Euglycemia group (n = 160)	P-value
Atrial fibrillation (%)	21 (8.2%)	9 (9.3%)	12 (7.5%)	0.78
Thrombolytic treatment (%)	92 (35.9%)	27 (28.1%)	75 (46.8%)	0.004
Primary PCI (%)	78 (30.4%)	33 (34.3%)	45 (28.12%)	0.36
Conservative medical treatment (%)	103 (40.2%)	36 (37.5%)	77 (48.1%)	0.12
Bleeding (%)	8 (3.1%)	3 (3.1%)	5 (3.1%)	0.70
Post-PCI mortality (%)	7 (8.9%)	6 (18.8%)	1 (2.2%)	0.03
Post-thrombolytic mortality (%)	5 (5.4%)	3 (11.11%)	2 (2.6%)	0.21
Hospital mortality (%)	18 (7.0%)	12 (12.5%)	6 (3.7%)	0.015

Univariate analysis revealed that significant risk factors of mortality in patients with hyperglycemia on admission were age 60 years or older (odds ratio [OR], 5.63 [1.54-20.58]; p = 0.007), heart failure on admission (OR, 6.84 [1.85-25.22)]; p = 0.003), T2DM (OR, 4.14 [0.50-33.96]; p = 0.05), and presenting with renal failure (OR, 6.78 [1.74-26.42]; p = 0.009; Table 3).

**Table 3:** Univariate analysis of predictors of in-hospital mortality in patients with hyperglycemia at admission CI, confidence interval.

Predictor	Odds ratio (95% CI)	P-value
Female gender	0.37 (0.10-1.35)	0.15
Age ≥60 years	5.63 (1.54-20.58)	0.007
Hypertension	0.81 (0.22-2.91)	0.50
Diabetes mellitus	4.14 (0.50-33.96)	0.05
Smoking	0.51 (0.15-1.74)	0.35
Heart failure on admission	6.84 (1.85-25.22)	0.003
Cardiogenic shock	7.54 (0.43-129.4)	0.23
Renal failure	6.78 (1.74-26.42)	0.009

## Discussion

In 2013, the European Society of Cardiology and the European Association for the Study of Diabetes issued guidelines for glycemic control in patients with cardiac disease with major goals to improve management in STEMI patients [9]. In the present study, we provide an estimation of the burden of STEMI patients with hyperglycemia and the associated risks for in-hospital mortality. Hyperglycemia with STEMI is a powerful predictor of poor in-hospital outcomes [10]. We found that STEMI patients with hyperglycemia develop more complications such as heart failure, cardiogenic shock, and atherosclerosis compared to euglycemic STEMI patients. While modern anti-hyperglycemic agents are effective in the treatment of elevated blood glucose levels, these drugs also affect the cardiovascular system [11-13].

In our study, hyperglycemic STEMI patients at the time of hospital admission had a higher mortality rate than euglycemic STEMI patients, even after reperfusion. Hyperglycemia’s impact on in-hospital outcomes does not seem to depend on the reperfusion strategy. According to Planer et al., hyperglycemia caused cardiac crises of myocardial re-infarct and bleeding following PCI [14]. Malmberg et al. reported that in STEMI patients stabilized by thrombolysis, hyperglycemia was associated with a higher mortality rate compared with euglycemic patients [5]. A Japanese study reported that hyperglycemia in patients with STEMI was associated with higher morbidity of large infarcts and higher in-hospital mortality compared with euglycemic patients [15]. STEMI outcomes in patients with T2DM can be improved after controlling blood glucose levels [16].

The significant risk factors of in-hospital mortality in hyperglycemia patients, according to our findings, were patient age of ≥60 years, T2DM, heart failure, and renal failure. Jomaa et al. reported similar risk factors such as age ≥75 years, anemia, heart failure, cardiogenic shock, renal failure, and bleeding as independent predictors of mortality in T2DM patients presenting with hyperglycemia [17].

Limitation of the study

Our study was limited in that the sample size was small. Future similar studies should be conducted in a large center with a larger study population to confirm cardiac outcomes of STEMI patients with hyperglycemia at admission.

## Conclusions

Hyperglycemia is independently associated with cardiac death in STEMI patients, and the predictive value of hyperglycemia is higher in T2DM patients compared to patients without T2DM. Hyperglycemia with STEMI has been a challenge for cardiologists around the world, and better management with effective treatment is the only strategy to decrease the morbidity and mortality rate.
